# Efavirenz-induced gynecomastia in a prepubertal girl with human immunodeficiency virus infection: a case report

**DOI:** 10.1186/1471-2431-13-120

**Published:** 2013-08-13

**Authors:** Mette S van Ramshorst, Magdeline Kekana, Helen E Struthers, James A McIntyre, Remco PH Peters

**Affiliations:** 1 Anova Health Institute, Khutso Kurhula Project, Johannesburg, South Africa; 2 Department of Internal Medicine, University of Cape Town, Cape Town, South Africa; 3 School of Public Health & Family Medicine, University of Cape Town, Cape Town, South Africa; 4 Anova Health Institute Khutso Kurhula Project, 21A Peace Street, P.O. Box 2243, Tzaneen, 0850, South Africa

**Keywords:** Gynecomastia, HIV, Efavirenz, Child, Prepubertal

## Abstract

**Background:**

Prepubertal gynecomastia is a rare condition and most frequently classified as idiopathic. In HIV-infected adults gynecomastia is a recognised but infrequent side-effect of antiretroviral treatment (ART) and mostly attributed to efavirenz use. Gynecomastia should be distinguished from pseudogynecomastia as part of the lipodystrophy syndrome caused by Nucleoside Reverse Transcriptase Inhibitors (NRTIs) to avoid incorrect substitution of drugs. In the medical literature only five cases of prepubertal gynecomastia in children taking ART are described and underlying pathogenesis was unknown. The occurrence of adverse effects of ART may interfere with therapy adherence and long-term prognosis and for that reason requires attention. We report the first case of prepubertal gynecomastia in a young girl attributed to efavirenz use.

**Case presentation:**

A seven-year-old African girl presented with true gynecomastia four months after initiation on ART (abacavir, lamivudine, efavirenz). History, physical examination and laboratory tests excluded known causes of gynecomastia and efavirenz was considered as the most likely cause. Six weeks after withdrawal of efavirenz the breast enlargement had completely resolved.

**Conclusions:**

Efavirenz-induced gynecomastia may occur in children as well as in adults. With the increasing access to ART, the possibility of efavirenz-exposure and the potential occurrence of its associated side-effects may be high. In resource-poor settings, empirical change from efavirenz to nevirapine may be considered, providing no other known or alarming cause is identified, as efavirenz-induced gynecomastia can resolve quickly after withdrawal of the drug. Timely recognition of gynecomastia as a side-effect of efavirenz is important in order to intervene while the condition may still be reversible, to sustain adherence to ART and to maintain the sociopsychological health of the child.

## Background

The clinical definition of gynecomastia is the presence of a rubbery or firm mass extending concentrically from the nipple of the breast, and the histological definition is the benign proliferation of glandular breast tissue. When the size of glandular tissue exceeds 0.5 cm in diameter gynecomastia can usually be palpated [1,2]. Gynecomastia should be distinguished from pseudogynecomastia which is defined as fat deposition without glandular proliferation, and is often seen in the presence of obesity [1,3,4]. Pseudogynecomastia has been reported in HIV-infected individuals taking antiretroviral treatment (ART) as part of the fat redistribution syndrome [1,5-7].

Most cases of gynecomastia in children occur in the neonatal period (due to placental transfer of estrogens) and in puberty (due to an imbalance between estrogens and androgens in the breast tissue); prepubertal gynecomastia is rare. Two studies show that only five percent of children with gynecomastia were prepubertal at diagnosis [3,8]. Isolated breast enlargement is usually referred to as prepubertal gynecomastia or premature thelarche [2,3,9]. Prepubertal gynecomastia should always be considered a pathological sign [2,3,10,11]. Although the majority of cases are idiopathic, there are various recognised causes of prepubertal gynecomastia including conditions of the liver, kidney, thyroid, adrenal glands and testicles (Table 1) [2,3,10,11]. In addition, various drugs have been associated with development of gynecomastia including antibiotics, neuropsychiatric medication and tuberculosis treatment [1-3,12,13]. Moreover, in adults, gynecomastia has been reported in association with most classes of antiretroviral agents and with Human Immunodeficiency Virus (HIV) infection itself [14,15]. There are very limited data on this condition in HIV-infected children on ART. We report on a prepubertal HIV-infected girl who developed gynecomastia shortly after initiation of ART. Publication of this case report was approved by the Human Ethics Research Committee of the University of the Witwatersrand, Johannesburg, South Africa (Ref: M130273).

**Table 1:** **Causes of prepubertal gynecomastia**[1-4,10,12]

**Type**	**Details**
Idiopathic	No known cause
Exposure to exogenous estrogens	• Dermal and hair preparations (e.g. lavender and tea tree oils)
	• Milk and meat of cows treated with estrogens
Illness	• Liver disease (e.g. cirrhosis)
	• Renal failure
	• Thyrotoxicosis
	• Malnutrition
Neoplasia	• Adrenal
	• Testicular
Congenital adrenal hyperplasia	• (Late-onset) 21-hydroxylase deficiency (21-OHD)
	• 11-beta-hydroxylase deficiency
	• Overexpression of aromatase
Other hormonal aberrations	• Androgen deficiency (e.g. congenital adrenal hyperplasia)
	• Androgen insensitivity
	• Extraglandular aromatization of androstenedione (e.g. aromatase excess syndrome (AES), obesity)
Medication	• Anti-androgens (e.g. bicalutamide, flutamide, finasteride, spironolactone)
	• Antimicrobials (e.g. antiretrovirals, isoniazid, ketoconazole, metronidazole, penicillamine)
	• Antineoplastic and immunomodulators (e.g. alkylating agents, bleomycin, cisplatin, cyclosporine, methotrexate, vincristine)
	• Gastrointestinal agents (e.g. cimetidine, metoclopramide, omeprazole, ranitidine)
	• Cardiovascular (e.g. amiodarone, amlodipine, angiotensin-converting enzyme inhibitors, calcium channel blockers, digitoxin, fibrates, reserpine, spironolactone, statins)
	• Hormones (e.g. androgens, chorionic gonadotropin, estrogens, GnRH agonist, GH)
	• Neurologic/psychiatric (e.g. haloperidol, methylphenidate, opioids, risperidone, TCAs)
	• Drugs of abuse (e.g. alcohol, amphetamines, heroin, marijuana, methadone)

*Abbreviations:GnRH* Gonadotropin-releasing hormone, *GH* Growth hormone, *TCAs* Tricyclic antidepressants.

## Case presentation

A 7-year old girl with HIV-infection presented at the ART clinic of a hospital in rural Mopani District, South Africa, with enlargement of both breasts since one month. She had been initiated on ART four months before and was taking a regimen of abacavir, lamivudine and efavirenz. She reported that the growth of her breasts occurred a few weeks earlier, approximately ten to twelve weeks after the start of ART. The breasts were slightly tender but otherwise painless, and the breasts had stabilized in size. She did not experience milky discharge from the nipple nor had she or her mother observed any signs that could be associated with early puberty such as menstruation, acne and pubic or axillar hair growth. Except from the breast enlargement she felt well and did not have any other complaints. She did not take any drugs of abuse or medication other than ART and co-trimoxazole prophylaxis with correct doses for her body weight.

Physical examination revealed bilateral glandular breast tissue, which could easily be distinguished from fat tissue through palpation. The breast gland tissue was about 4-5 cm in diameter, Tanner stadium 3, with the left breast being more prominent than the right. There was no discharge observed from the nipple. There were no signs of lipodystrophy or puberty, in particular no pubic or axillar hair growth. The body mass index (BMI) was 13.3 and BMI for age was below the 15th percentile. On clinical examination the thyroid gland was not enlarged.

Blood tests were performed for liver and kidney function and results were within the normal range. Blood hormone levels were all in the normal range as well and were as follows: thyroid-stimulating hormone (TSH) 5.12 mIU/L; testosterone < 0.35 nmol/L; cortisol 129 nmol/L; follicle-stimulating hormone (FSH) < 2 IU/L; luteinizing hormone (LH) < 0.5 IU/L; estradiol < 92.0 ρmol/L; prolactin 6.6 μg/L. The CD4 cell count was 302 cells/mm^3^ compared to 330 cells/mm^3^ at ART initiation; viral load was 174 copies/mm^3^. Baseline viral load was not available in accordance with South African treatment guidelines.

A clinical diagnosis of medication-induced gynecomastia was considered and efavirenz was replaced with nevirapine in her ART regimen (no history of previous nevirapine exposure). At two weeks follow-up the breast size was reduced by half to 2–3 cm in diameter and 4 weeks later the gynecomastia had resolved completely.

## Discussion and conclusion

The occurrence of adverse effects of ART is an important determinant of therapy adherence and therefore of long-term prognosis [16,17]. Gynecomastia is a recognised but rare side-effect of ART in adult patients with an estimated incidence of 2.8% in Western European male patients treated with ART for more than two years [18]. Another study from Europe showed a similar incidence of 2.9% (15 out of 516 patients) in male patients treated with different ART-regimens [7]. In HIV-infected children gynecomastia has only been reported on five occasions [7,19-21]. Manfredi and colleagues described three cases of gynecomastia confirmed by ultrasonography in boys aged 11 years (1) and 12 years (2) with congenitally acquired HIV disease [7,19]. These children were taking a regimen of stavudine and didanosine with a protease-inhibitor. No efavirenz exposure was reported and two children presented with concurrent mild lipodystrophy syndrome. Pubertal development was described as prepubertal for two patients and for the latter it was not described. The authors did not associate the development of gynecomastia with a specific drug. Another case report described the development of breasts in a 15 year old HIV-infected African boy two years after his regimen was changed from abacavir, lamivudine and nelfinavir to stavudine, didanosine and efavirenz because of virological failure. Gynecomastia was persistent despite withdrawal of efavirenz and eventually bilateral mastectomy was required. This case was an adolescent boy who already had gone through normal pubertal development before the development of bilateral gynecomastia [20]. Finally, a study from Uganda on occurrence of side-effects in children on ART reported one case of gynecomastia in a child taking zidovudine, lamivudine and efavirenz, but information on age, pubertal stage, follow-up and switch of regimen are not provided [21]. To our knowledge, we are the first to describe a case of gynecomastia in a prepubertal child that can be attributed to efavirenz.

Efavirenz was the most likely cause of gynecomastia in our case for several reasons. First, we could not identify a recognised physical cause of gynecomastia in this prepubertal girl through history, physical examination and laboratory tests. Precocious puberty was ruled out as no other signs of puberty were observed and gynecomastia resolved after intervention. This suggests that the gynecomastia should be classified as either medication-induced or idiopathic. Secondly, efavirenz is a recognised cause of gynecomastia in adults on ART in whom withdrawal of this drug from the ART regimen has been associated with regression of symptoms [6,22]. A similar clinical course is observed in our patient. Although gynecomastia has been reported for almost all antiretroviral drugs, efavirenz has been implicated far more frequently in adults [6,7,14,22,23]. Finally, the time course of the complication is suggestive of an association with efavirenz although this does not prove causality: gynecomastia occurred relatively quickly after introduction of the drug, the time period between initiation of ART and onset of symptoms (approximately 3 months) is similar to that described for adults, and the gynecomastia resolved quickly after withdrawal of the drug [6,22,23]. The resolution of gynecomastia upon withdrawal of efavirenz in our child (6 weeks) was much quicker than reported for spontaneous resolution of idiopathic gynecomastia (0.8 – 2.6 years) in a case series of 27 children with idiopathic gynecomastia (not all prepubertal) of whom six showed spontaneous resolution [3]. Altogether, we think that efavirenz is the most likely causative agent of gynecomastia in our patient. However we refrained from reintroducing efavirenz, which could provide more substantiation of a causal association.

This hypothesis is supported by a plausible pathological pathway of how efavirenz may induce gynecomastia. The interaction of efavirenz with breast tissue causing breast enlargement and increased risk of malignancy may be indirect or direct. Sikora and colleagues demonstrated direct binding of efavirenz to the estrogen-receptor-alpha in the breast [24]. This receptor induces cell growth in an 17β-oestradiol-dependent breast cancer model. A different theory relates to gynecomastia occurring as an immune restoration phenomenon whereby cytokine and hormonal balances are disturbed [14]. The latter theory does not apply to our case as there was no change in CD4 count observed since initiation of ART and viral load was not yet fully suppressed.

The natural course of efavirenz-associated gynecomastia is under debate in the literature. One case study described spontaneous complete resolution in 12 out of 15 patients (80%) after a mean period of two months without any specific therapy [14]. Another study showed only partial spontaneous regression of breast hypertrophy in two out of six patients (33%) without treatment modification [23]. A case series described persistence of gynecomastia in three patients without intervention, with one patient requiring mastectomy [22]. Manfredi and colleagues reported stabilisation of breast development when efavirenz was continued [7]. Finally one study reported withdrawal of efavirenz as intervention with complete resolution of gynecomastia in all five patients in a mean period of five months [6].

Recognition of gynecomastia as a side-effect of efavirenz use is important with efavirenz being a preferred component of first-line treatment regimen of HIV-infection worldwide. Taking into consideration the increasing access to ART, the potential for efavirenz-exposure and its associated side-effects is large [25]. Furthermore the occurrence of side-effects is known to be the most frequent reason of discontinuation of at least one drug in the initial regimen in the first year after ART initiation [17]. Socio-psychological affliction is another major reason to manage this side-effect intensively. Young children, especially boys, with breast development have been reported to suffer from cultural and social stigma [3,4,20,26,27]. In addition prepubertal children with gynecomastia may be considered sexually mature at too young an age. Timely recognition of drug-induced gynecomastia offers the possibility of non-invasive intervention because the condition is reversible if recently developed, but substantial regression is unlikely if existent for more than a year as fibrotic tissue is usually present [1]. As described previously the differential diagnosis of gynecomastia is broad and the most important diagnosis to be ruled out is a breast malignancy. A meta-analysis of the incidence of non-AIDS-defining cancers in HIV-infected patients showed that the incidence of breast cancer has significantly increased since the implementation of ART as standard therapy [28]. It remains to be seen if the estrogenic activity of efavirenz may promote breast cancer.

Although gynecomastia may be rare, it is important to educate healthcare workers about this condition and on how to differentiate between gynecomastia and lipodystrophy on physical examination. This distinction is important to avoid incorrect substitution of drugs as lipodystrophy is usually caused by nucleoside reverse transcriptase inhibitors (NRTIs). In resource-poor settings in Africa where diagnostic facilities are not available, an empirical switch from efavirenz to nevirapine may be considered providing history, physical examination and basic laboratory tests do not reveal a known cause. The substitution of nevirapine for efavirenz is considered safe and maintains virological suppression in most patients [29]. In addition nevirapine-based regimens show comparable activity to efavirenz-based regimens [25].

In conclusion, this case report shows that efavirenz may induce gynecomastia in prepubertal HIV-infected children on ART with complete resolution after withdrawal of efavirenz. Early recognition and differentiation from lipodystrophy are important to timely and correctly manage this side-effect in order to improve health, sustain adherence to ART and to reduce psychosocial stigma.

### Consent

Written informed consent was obtained from the mother of the patient for publication of this case report. A copy of the written consent is available for review by the Editor of this journal.

## Abbreviations

21-OHD: 21-Hydroxylase deficiency; AES: Aromatase excess syndrome; ART: Antiretroviral treatment; BMI: Body mass index; FSH: Follicle-stimulating hormone; GnRH: Gonadotropin-releasing hormone; GH: Growth hormone; HIV: Human immunodeficiency virus; LH: Luteinizing hormone; NRTIs: Nucleoside Reverse Transcriptase Inhibitors; TCAs: Tricyclic antidepressants; TSH: Thyroid-stimulating Hormone.

## Competing interests

None of the authors reports competing interests.

## Authors’ contributions

MSvR contributed to the acquisition and interpretation of data, has made substantial contributions to conception and design and has been involved in drafting the manuscript. MK contributed to the acquisition of data and commented on the manuscript. HES revised the manuscript critically for important intellectual content. JAM revised the manuscript critically for important intellectual content. RPHP contributed to the acquisition and interpretation of data, has made substantial contributions to conception and design and has been involved in drafting the manuscript. All authors read and approved the final manuscript to be published.

## Pre-publication history

The pre-publication history for this paper can be accessed here:

http://www.biomedcentral.com/1471-2431/13/120/prepub
